# Water Harvesting Performance of Modified Nanostructure Aluminum Using Silica Nanoparticles Coating and Laser Processing

**DOI:** 10.3390/nano15110828

**Published:** 2025-05-29

**Authors:** Milin Lekmuenwai, Piyachit Yingkiatinon, Warin Namkotr, Chatchawan Tancharoensup, Tanyakorn Muangnapoh, Tippawan Sodsai, Paiboon Sreearunothai, Krissada Surawathanawises, Bhawat Traipattanakul

**Affiliations:** 1 School of Integrated Science and Innovation, Sirindhorn International Institute of Technology, Thammasat University, Pathum Thani 12120, Thailandpaiboon_sree@siit.tu.ac.th (P.S.); 2 School of Manufacturing Systems and Mechanical Engineering, Sirindhorn International Institute of Technology, Thammasat University, Pathum Thani 12120, Thailand; 3 National Nanotechnology Center (NANOTEC), National Science and Technology Development Agency (NSTDA), Pathum Thani 12120, Thailand; tanyakorn.mua@nanotec.or.th (T.M.);; 4 Department of Materials Engineering, Faculty of Engineering, Kasetsart University, Bangkok 10900, Thailand; fengksds@ku.ac.th

**Keywords:** hydrophobic, superhydrophobic, hydrophilic, biphilic, silica nanoparticles, nanolaser, aluminum, condensation, water harvesting

## Abstract

Dew collection is one of the most efficient water harvesting methods. In this work, we experimentally investigated the effects of modified nanostructured surfaces on water harvesting performance. Aluminum surfaces exhibiting hydrophobic, superhydrophobic, hydrophilic, and biphilic properties were utilized in this study. The superhydrophobic surface was fabricated using a fluorinated modified silica nanoparticles coating, while nanolaser processing and the surface abrasion with sandpapers were employed to create two distinct hydrophilic structures. In addition, various biphilic surface patterns, incorporating both superhydrophobic and hydrophilic characteristics, were also fabricated. The nanolaser-treated surface demonstrated the highest water harvesting performance, achieving a water collection of 386.7 mL/m^2^. This performance represented a 42% increase compared to unpolished sample and a 282% increase relative to the superhydrophobic sample. Furthermore, the results indicated that the optimal biphilic surface pattern occurred at a 1:4 superhydrophobic-to-hydrophilic area ratio. The experimental outcomes were further interpreted through the mechanisms underlying water harvesting. Additionally, the experimental results were explained with the water harvesting mechanism.

## 1. Introduction

Water is fundamental to life and is utilized across all sectors. However, only about 1% of water on the Earth’s surface is accessible freshwater [1,2]. This limited freshwater supply is further strained by drought, population growth, unsustainable usage, and climate change, particularly in equatorial regions [3,4]. To address these challenges, various water harvesting methods have been developed. Common techniques include rainwater harvesting, surface runoff harvesting, fog harvesting, and groundwater recharge. However, rainwater harvesting depends on seasonal availability, while surface runoff harvesting relies heavily on annual rainfall intensity. In addition, groundwater recharge is constrained by the geological characteristics of the area, and fog collection is only effective in regions with high fog density [5,6,7,8]. Therefore, this study proposes dew collection using modified nanostructured solid metal surfaces. This approach offers a cost-effective and environmentally friendly solution which can operate under a wide range of climatic conditions, making it suitable for deployment in most regions.

Water harvesting via dew collection on solid surfaces occurs through the process of condensation. Condensation can occur in a bulk state as a homogeneous process and on a cooled surface as a heterogeneous process [9,10]. In addition to ambient conditions, the efficiency of condensation is influenced by the structure of the surface [11,12]. Variations in surface structure lead to differences in surface properties, affecting key parameters such as droplet adhesion, mobility, and coalescence behavior [13]. On a cooled surface, condensation manifests in two modes: dropwise condensation and film-wise condensation [14,15]. One of the key surface characteristics affecting condensation performance and droplet dynamics is surface wettability, which is defined by the water contact angle. Surfaces with contact angles below 10° are classified as superhydrophilic, while those with angles between 10° and 90° are considered hydrophilic. In contrast, hydrophobic surfaces exhibit water contact angles greater than 90°. A surface is deemed superhydrophobic when the contact angle exceeds 150° and the sliding angle is less than 10°. Such surfaces exhibit high water repellency due to their extremely low surface energy and offers beneficial properties including self-cleaning, anti-icing, anti-fogging, and heat transfer enhancement [16,17,18,19,20,21,22]. It is important to note that while hydrophilic and hydrophobic surfaces can occur naturally in common substrate materials, specialized fabrication techniques are required to achieve low-contact-angle hydrophilic, superhydrophilic, and superhydrophobic surfaces [23,24]. In addition, biphilic surface combines hydrophilic and hydrophobic regions, designed to harness the advantages of both properties [25].

Surfaces with modified wettability have been widely applied in water harvesting and various engineering applications. Bai et al. (2014) [26] modified a glass surface with TiO_2_ slurry to create star-shaped wettability patterns. The star-shaped patterns significantly enhanced water collection, achieving water harvesting rate ranging from 2.11 to 2.78 g/cm^2^ per hour. Liu et al. (2020) [27] developed a surface with superhydrophobic and hydrophobic regions infused with lubricating oil. This design achieved a collection efficiency of up to 3%, surpassing the performance of conventional systems. Zhang et al. (2021) [28] developed a durable superhydrophobic Ti6Al4V surface through a multistep process involving lithography, acid-etching, anodizing, and fluorination. The surface demonstrated effective fog collection under a relative humidity condition of 90%. Knapczyk-Korczak et al. (2020) [29] modified commercial Raschel mesh with electrospun polyamide 6 (PA6) nanofibers. The modified mesh collected three times more water than the unmodified mesh, achieving a water harvesting rate of 64 mg/cm^2^/h under controlled laboratory conditions. Cheng et al. (2021) [30] fabricated a Rhacocarpus-inspired porous surface (RIPS) on a silicon substrate using microelectromechanical systems technology. The RIPS exhibited a three-level wettability gradient, with contact angles of 80.9° on the top, 66° on the sidewalls, and 45.6° on the bottom of the holes. Under the temperature of 43 °C and the relative humidity of 91%, the RIPS achieved a 160% improvement in water harvesting compared to conventional hydrophobic surfaces. Wang et al. (2015) [31] employed a simple and low-cost thermal pressing technique to fabricate patterned hybrid surfaces by combining hydrophilic polystyrene (PS) sheets with superhydrophobic copper gauze. Experimental results showed that these hybrid surfaces achieved significantly higher fog collection rates compared to surfaces that were purely hydrophilic or superhydrophobic. Gou et al. (2020) [32] developed a hierarchical CuO@TiO_2_-coated surface featuring alternating hydrophilic and hydrophobic patterns. Under the relative humidity of 90%, these patterned surfaces demonstrated enhanced fog collection efficiency relative to uniformly superhydrophobic or superhydrophilic surfaces. Recent work by Wong et al. (2023) showed that superhydrophobic surfaces with optimized microflower structures (3–5 μm) improved atmospheric water harvesting by enhancing droplet nucleation and promoting efficient droplet departure [33]. Wei et al. (2024) introduced triangular patterns to superhydrophilic surfaces to prevent water puddle formation, thereby improving dew harvesting performance. The most effective patterned design increased water collection efficiency by 78% compared to non-patterned superhydrophilic surfaces and by 576% compared to non-patterned superhydrophobic surfaces [34].

Despite extensive studies on water harvesting, a comprehensive understanding of how surfaces with varying wettabilities perform in such applications remain limited. To address this gap, the present research focuses on the development and fabrication of modified surfaces—hydrophilic, superhydrophobic, and biphilic—on aluminum substrates. Superhydrophobic surfaces were created using silica nanoparticles functionalized with Tridecafluorooctyl triethoxy silane (FAS). Two types of hydrophilic structures were fabricated through nanolaser treatment and sandpaper abrasion. Furthermore, a variety of nanostructured biphilic surfaces with different wettability patterns were developed. Surface characterization was conducted to assess the morphological and wettability features on the modified substrates. This influence of surface structure on water harvesting performance was experimentally investigated and compared under a controlled ambient condition. Notably, this study is the first to investigate the use of modified wettability surface structures on aluminum substrates for water harvesting applications.

## 2. Materials

### 2.1. Materials Preparation

Aluminum was selected as the substrate material for this work. Aluminum plates measuring 50 × 50 mm with a thickness of 1 mm were used. Five distinct types of surface samples were prepared: unpolished, polished, coated, lasered, and coated and lasered patterned. The unpolished samples were prepared by cleaning the aluminum plates with isopropyl alcohol (IPA), followed by placement in an ultrasonic bath for 5 min. For the polished, coated, lasered, and coated and lasered patterned samples, the aluminum plates were polished using sandpaper with grid sizes of 600, 1200, and 2000 in sequence. After polishing, the aluminum plates were cleaned with IPA and placed in an ultrasonic bath for 5 min. At this stage, the polished samples were obtained. To fabricate a superhydrophobic structure on the aluminum samples, a fluorinated modified silica nanoparticle solution was prepared. The solution comprised 50 mL of tetraethylorthosilicate (TEOS), 434 mL of isopropyl alcohol, 16 mL of ammonium hydroxide, and 5 mL of tridecafluorooctyl triethoxy silane (FAS) [16,17]. This mixture was used to synthesize fluorinated silica nanoparticles. The prepared solution was then sprayed onto the polished and cleaned aluminum surfaces, yielding the coated samples. A 2 mL of mixture solution was used for the coating process. During spray coating, the distance between the spray nozzle and the sample was kept at 15 cm. After coating, the sample was maintained at 25 °C for 10 min to complete the fabrication of the coated surface. To fabricate the lasered and coated and lasered patterned samples, the Fiber Laser Marking Machine (M-20, Qiuye Numerical Control Technology (Shanghai) Co., Ltd, Shanghai, China) was employed with the following parameters: 0.1 mm line spacing, 80% power, 500 mm/s scanning speed, 20 kHz frequency, a wavelength of 1064 nm, and a pulse duration of 100 ns. The lasered samples were produced by applying nanolaser processing across the entire surface of the coated aluminum plates. For the coated and lasered patterned samples, patterned surfaces were created on the coated plates by alternating coated and lasered regions with varying area ratios of 1:1, 1:2, 1:3, 1:4, 1:5, and 1:6. The width of the coated region was fixed at 0.5 mm, while the width of the adjacent lasered regions was varied from 0.5 mm to 3 mm accordingly. As a result, the coated and lasered patterned samples consisted of both coated and lasered regions arranged in defined patterns. It is crucial to note that, since nanolaser processing was applied to the coated surfaces of the coated and lasered patterned samples, the lasered samples were also coated prior to laser treatment. This approach ensures consistency in surface preparation and enabled a fair comparison between the two sample types.

### 2.2. Surface Characterization

A comprehensive set of characterization techniques was employed to analyze the modified nanostructured surfaces in this study. These techniques provide a detailed understanding of their chemical and physical properties. A Mobile Surface Analyzer (KRÜSS GmbH, Hamburg, Germany) was used to measure water contact angles and surface energy values. A droplet of 2 µL was used for the water contact angle measurement. To determine surface energy, 2 µL of both water and diiodomethane were used as probe liquids, and the total surface energy, along with its polar and dispersive components, was calculated by the instrument using the Owens–Wendt method. Also, advancing and receding contact angles were measured by injecting and withdrawing water at a controlled rate of 1 µL/s using a contact angle instrument (Dataphysics OCA40, DataPhysics Instruments GmbH, Filderstadt, Germany). The contact angle hysteresis is calculated by subtracting the receding contact angle from the advancing contact angle. For each surface parameter mentioned above, three measurements were performed at three different locations on each sample. The average values of water contact angles, surface energy, advancing and receding contact angles, and the calculated contact angle hysteresis were reported. In addition, a confocal microscope (OLYMPUS DSX1000, Evidence Corporation, Tokyo, Japan) was used to measure the surface roughness of the fabricated surfaces. Additionally, the surface morphology of the fabricated surfaces was examined using scanning electron microscopy (SEM, FE-SEM SU8230, Hitachi High-Technologies Corporation, Tokyo, Japan). Furthermore, to analyze the chemical compositions of the surfaces of the coated and lasered samples, energy-dispersive spectroscopy (EDS, FE-SEM SU8230, Hitachi High-Technologies Corporation, Tokyo, Japan) was employed.

## 3. Experimental Setup

**Figure 1:**
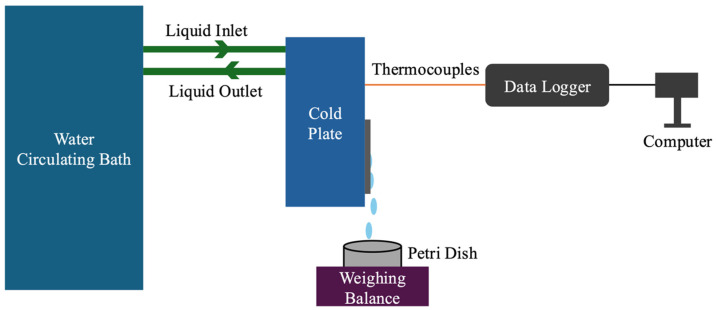
A schematic diagram of the water harvesting experiment.

As shown in Figure 1, a cold plate was composed of an aluminum plate integrated with a Teflon block. The aluminum plate measured 11 × 11 × 3 cm and featured an internal liquid circulation channel with a diameter of 8 mm. This channel equipped with one inlet port and one outlet port facilitated the flow of a cooling liquid through the plate. The Teflon block, with dimensions of 16 × 16 cm and a thickness of 5 cm, was machined to include a rectangular slot measuring 11 × 11 cm with a depth of 3 cm. The aluminum plate was fitted into this slot, ensuring close contact between the components. Due to the low thermal conductivity of Teflon, heat loss was effectively minimized. A water-circulating bath equipped with a temperature controller was connected to the cold plate via the inlet and outlet ports of the Teflon block and the aluminum plate, providing the cooling power. A mixture solution of ethylene glycol and water was used as the circulating fluid to maintain consistent thermal conductivity and prevent freezing. Thermocouples were attached to the cold plate to monitor surface temperature, which was maintained at 7 °C throughout the experiment. The test sample was mounted directly onto the surface of the cold plate, and thermal grease was applied at the interface to enhance thermal contact. To reduce condensation in non-essential areas, the exposed regions of the aluminum plate not covered by the test sample were insulated using thermal tapes. The cold plate was positioned vertically at a 90-degree angle, aligned parallel to the direction of gravity. A Petri dish and a weight balance were placed beneath the test sample to collect and measure condensed water droplets. The mass of the collected water was recorded at 20 min intervals over a total test duration of 4 h per sample. Each sample was tested three times to ensure repeatability and reduce experimental error. All experiments in this study were conducted in a controlled environmental chamber maintained at 25 °C ambient temperature and 60% relative humidity. The uncertainties in temperature and humidity control were at ±0.7 °C and ±2%, respectively. The chamber remained sealed throughout each test to ensure consistent environmental conditions.

## 4. Results and Discussion

### 4.1. Surface Characteristics

**Figure 2:**
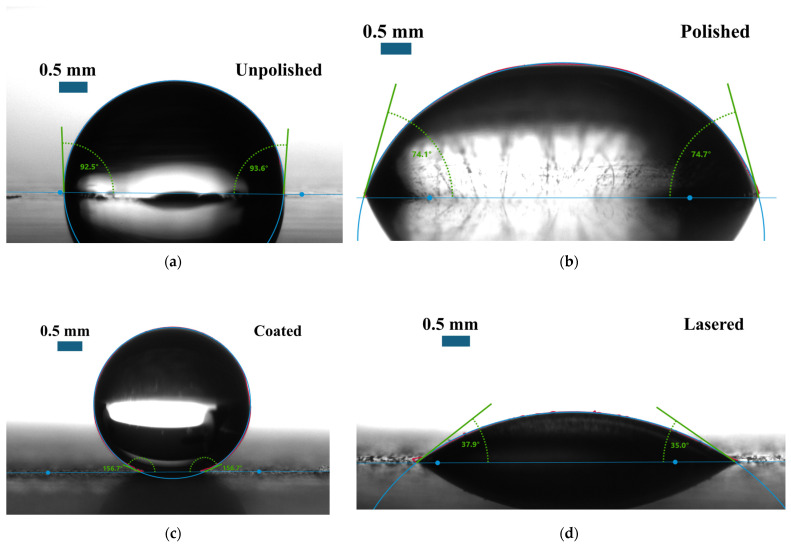
Water contact angle measurements of (**a**) the unpolished sample, (**b**) the polished sample, (**c**) the coated sample, and (**d**) the lasered sample.

**Figure 3:**
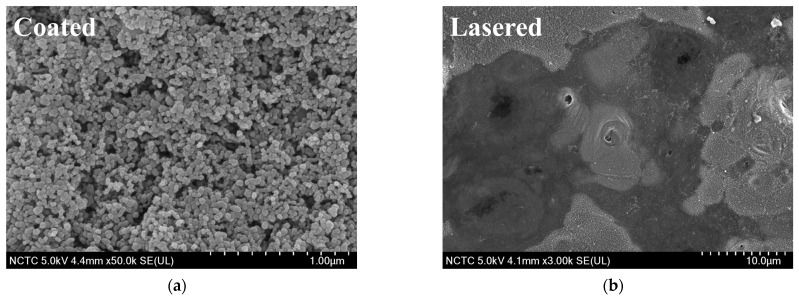

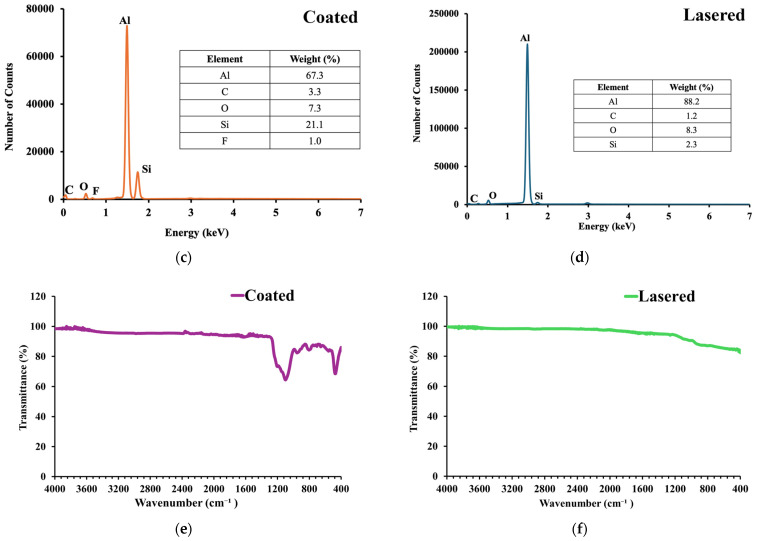
Surface characterization of the samples. (**a**) SEM image of the coated sample, (**b**) SEM image of the lasered sample, (**c**) EDS analysis of the coated sample, (**d**) EDS analysis of the lasered sample, (**e**) FTIR result of the coated sample, and (**f**) FTIR result of the lasered sample.

As shown in Table 1, the average water contact angles measured on the surfaces of the unpolished, polished, coated, and lasered samples were 94.1 ± 1.0°, 77.7 ± 1.2°, 157.0 ± 1.5°, and 35.9 ± 1.4°, respectively. Figure 2 shows the contact angle measurement of each surface type. The unpolished surface was hydrophobic, while the surface of the polished sample was hydrophilic. In addition, the superhydrophobic surface was successfully fabricated on the coated sample with the deposition of the silica nanoparticles. Nevertheless, when the coated surface was treated with nanolaser, the lasered surface with the hydrophilic property was achieved. It should be noted that the surface of the coated and lasered patterned sample possessed biphilic characteristics since they consisted of both coated and lasered areas. In this context, both superhydrophobic and hydrophilic properties were present on the surface of the coated and lasered patterned sample. In addition to the water contact angle results, the averaged surface energy values were also reported. The surface energy value of the coated sample was lowest at 0.4 ± 0.1 mN/m. On the other hand, the lasered surface exhibited the maximum surface energy of 78.9 ± 1.3 mN/m. While the surface energy of the unpolished sample was recorded at 21.1 ± 1.9 mN/m, the value at 33.5 ± 0.8 mN/m was obtained for the polished surface. The results of the water contact angle aligned with those of the surface energy. A lower surface energy correlated with a higher contact angle and vice versa. Surface roughness analysis revealed that the surface of the unpolished sample exhibited the greatest roughness due to the presence of contaminants and its random micro-level surface morphology. Although the coated sample exhibited the highest contact angle due to its superhydrophobic surface, its surface roughness was lower than that of the unpolished sample. This phenomenon can be attributed to the presence of silica nanoparticles, which enhanced the surface hydrophobicity at the nanoscale. In addition, compared to the unpolished sample, the reduction in surface roughness of the polished sample was achieved through simple sandpaper abrasion. Lastly, laser processing smoothed the surface of the lasered sample, leading to the lowest surface roughness among all samples. The contact angle hysteresis, defined as the difference between the advancing and receding angles, was averaged and is reported in Table 1. The unpolished, polished, coated, and lasered samples showed the contact angle hystereses of 17.7 ± 0.2°, 20.5 ± 0.2°, 6.9 ± 0.3° and 4.5 ± 0.3°, respectively. The SEM images shown in Figure 3a and Figure 3b illustrate the surface morphologies of the coated sample and the lasered sample, respectively. The silica nanoparticles remained distributed and intact on the coated sample following the coating process. In contrast, the laser treatment effectively removed the coating layer from the surface of the lasered sample, resulting in a significantly altered morphology. Notably, interfacial lines separating the coated and the laser-treated regions were observed on the surface of the coated and lasered patterned sample. As depicted in Figure 3c,d, silicon (Si) and fluorine (F) originated from the TEOS-FAS superhydrophobic solution were detected on the coated sample. For the lasered surface, only a minimal trace of Si was detected after the laser treatment. This indicates the effective removal of the fluorinated silica nanoparticle layer following the laser treatment. These combination results were also observed on the coated and lasered patterned sample with different elemental compositions corresponding to the coated and laser-treated regions. As shown in Figure 3e, the FTIR results for the coated sample exhibited characteristic peaks at 1203 cm^−1^, 1140 cm^−1^, 1068 cm^−1^, 954 cm^−1^, 657 cm^−1^, 472 cm^−1^, corresponding to the –CF_2_ asymmetrical stretching mode, the –CF_2_ symmetrical stretching mode, the Si–O–Si asymmetric stretching, the Si-F bond, the –CF2 wagging mode, and the Si–O–Si bending vibrations, respectively [17,35,36,37]. In addition, Figure 3f shows a peak at 419 cm^−1^, corresponding to Al–O–C bending vibrations [38].

### 4.2. Water Harvesting Performance

**Figure 4:**
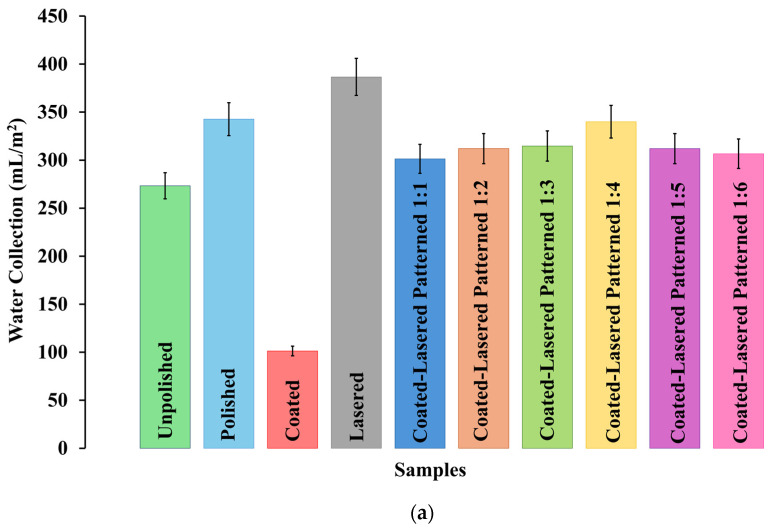

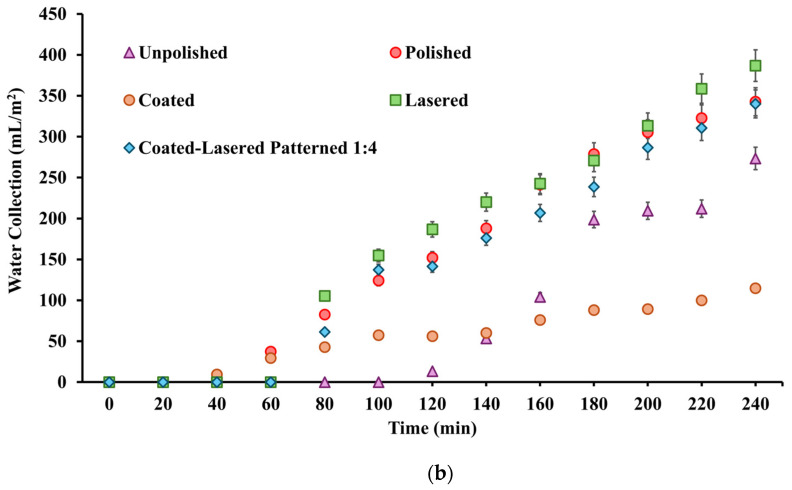
Water harvesting performance of all sample types. (**a**) Total water harvesting performance over a four-hour period. (**b**) Time-dependent water harvesting performance.

Figure 4a shows a comparative analysis of water harvesting performance over the four-hour experiment for all sample types: unpolished, polished, coated, lasered, and coated and lasered patterned. The unpolished sample, which featured a hydrophobic surface, exhibited moderate water collecting performance, accumulating 273.3 mL/m^2^ over the test duration. The polished sample demonstrated improved performance, collecting 342.7 mL/m^2^ of water—an increase of 25% compared to the unpolished sample. In contrast, the coated sample, characterized by its highly water-repellent superhydrophobic surface, recorded the lowest water collection at just 101.3 mL/m^2^. This represented a substantial reduction in performance—63% and 70% lower than the unpolished and polished samples, respectively. The lasered sample, modified through nanolaser treatment, achieved the highest water harvesting performance of all samples. It collected 386.7 mL/m^2^ of water, representing increases of 42%, 13%, and 282% compared to the unpolished, polished, and coated samples, respectively. The water harvesting results for the coated and lasered patterned samples with varying ratios of superhydrophobic to hydrophilic areas revealed a non-linear trend. Initially, as the proportion of hydrophilic area increased, water collection improved. The 1:1 coated and lasered patterned sample collected 301.3 mL/m^2^, while the 1:4 coated and lasered patterned sample achieved the highest performance at 340.0 mL/m^2^. This represented increases of 13%, 24%, and 236% compared with the 1:1 coated and lasered patterned, unpolished, and coated samples, respectively. Notably, the 1:4 ratio corresponded to superhydrophobic and hydrophilic widths of 0.5 mm and 2 mm, respectively. However, when the ratio was greater than 1:4, the water harvesting performance declined. The 1:5 and 1:6 coated and lasered patterned samples collected 312.0 mL/m^2^ and 306.7 mL/m^2^, respectively, indicating that a 1:4 ratio represents the optimal balance for maximizing condensation performance. Although the experimental results showed comparable water harvesting results between the polished and coated and lasered patterned samples, it is important to highlight the added functional advantages of the coated and lasered patterned samples due to their biphilic surface characteristics. In addition to the water harvesting performance reported in this study, previous research has shown that biphilic surfaces can also enhance heat transfer [39,40], and provide additional benefits such as self-cleaning [41,42], anti-fogging, and anti-icing properties [43,44].

**Figure 5:**
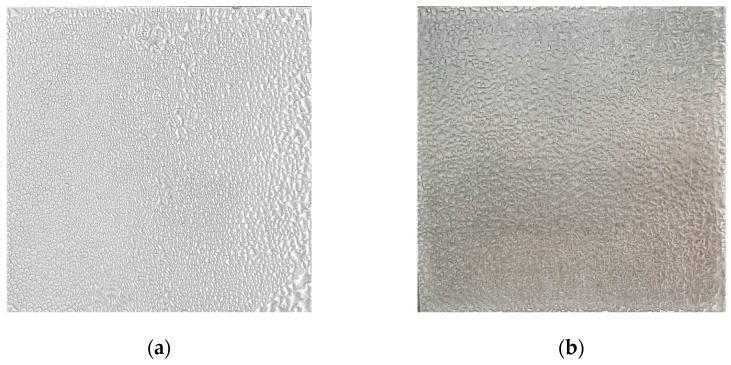

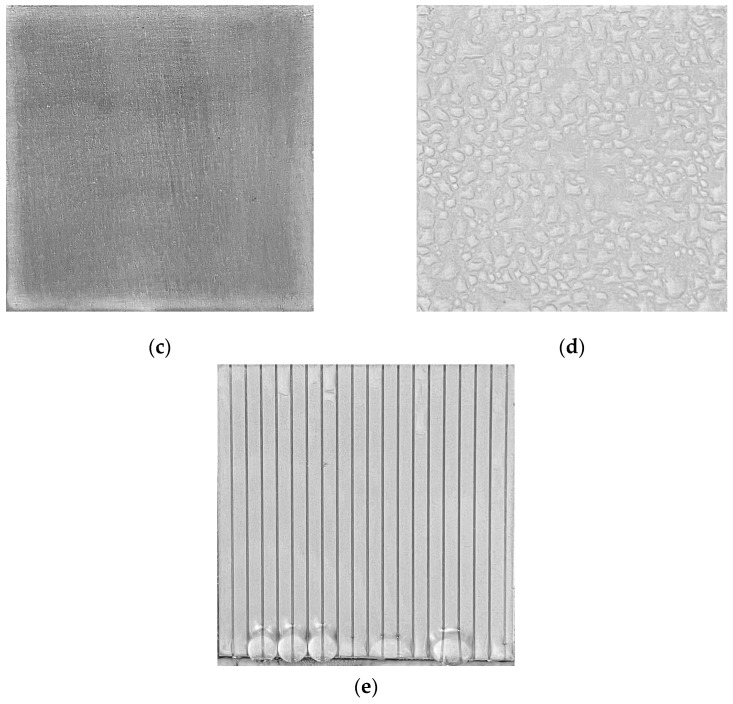
Water condensation behavior observed on (**a**) the unpolished sample, (**b**) the polished sample, (**c**) the coated sample, (**d**) the lasered sample, and **(e**) the coated and lasered patterned 1:4 sample.

Figure 4b illustrates the time-dependent water harvesting performance for all samples. The unpolished sample exhibited the longest delay in droplet formation and detachment, with the first water collection observed at the 120th minute. As shown in Figure 5a, droplets condensed on the unpolished sample were small in size, remained pinned, and did not merge with nearby droplets. When the surface was covered with water, the next cycle of condensation delayed. For the polished sample, no water was collected during the first 40 min, with the first droplet detachment at the 60th minute. In contrast, the coated sample, owing to its superhydrophobic nature, initiated condensation more rapidly. On this surface, the condensed droplets readily coalesced with neighboring droplets and detached from the surface in a random manner. Many droplets did not fall directly into the Petri dish, significantly reducing the amount of collected water. As illustrated in Figure 5c, a very small number of droplets remained on the coated surface. The lasered sample demonstrated a more uniform water distribution across its surface, as shown in Figure 5d. Between the 60th and the 80th minutes, the large coalesced droplets mitigated downward into the Petri dish. The cleared surface regions enabled continuous condensation, contributing to the high overall water collection performance. For the 1:4 coated and lasered patterned sample, water collection began after one hour. As shown in Figure 5e, the biphilic nature of this surface promoted efficient condensation. Water accumulated on the hydrophilic regions, while the adjacent superhydrophobic areas facilitated droplet coalescence and transport. This synergistic behavior enhanced water harvesting performance relative to the coated and unpolished samples.

**Figure 6:**
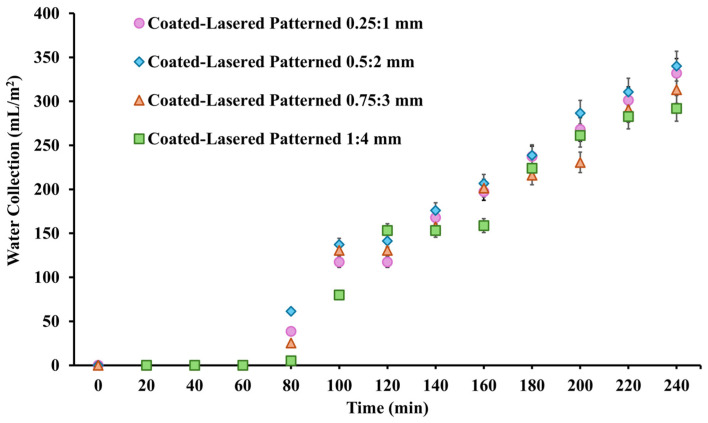
Water harvesting performance of the 1:4 coated and lasered patterned samples with various coated-to-lasered width configurations.

To obtain a more comprehensive understanding of the effects of the coated and lasered region widths on water harvesting performance, additional variants of the 1:4 coated and lasered patterned samples were fabricated, experimentally investigated, and compared. The fabrication processes followed the procedures outlined in Section 2, with modifications made to both the superhydrophobic and hydrophilic region widths. The coated and lasered patterned samples with coated-to-lasered width configurations of 0.25:1 mm, 0.5:2 mm, 0.75:3 mm, and 1:4 mm were fabricated. Figure 6 showed that the different width configurations impacted water harvesting performance. The optimal configuration—0.5 mm superhydrophobic width and 2 mm hydrophilic width—achieved the highest water collection, with a water harvesting performance of 340 mL/m^2^. When the width of the superhydrophobic region decreased to 0.25 mm, the water collection slightly dropped by approximately 2%. Conversely, as the superhydrophobic width increased to 0.75 mm and 1 mm, the performance declined by 8% and 14%, respectively. Although the water collection values from different coated to lasered width configurations overlapped, the results suggest that changes in the widths lead to corresponding changes in water harvesting performance.

### 4.3. Water Harvesting Mechanism

**Figure 7:**
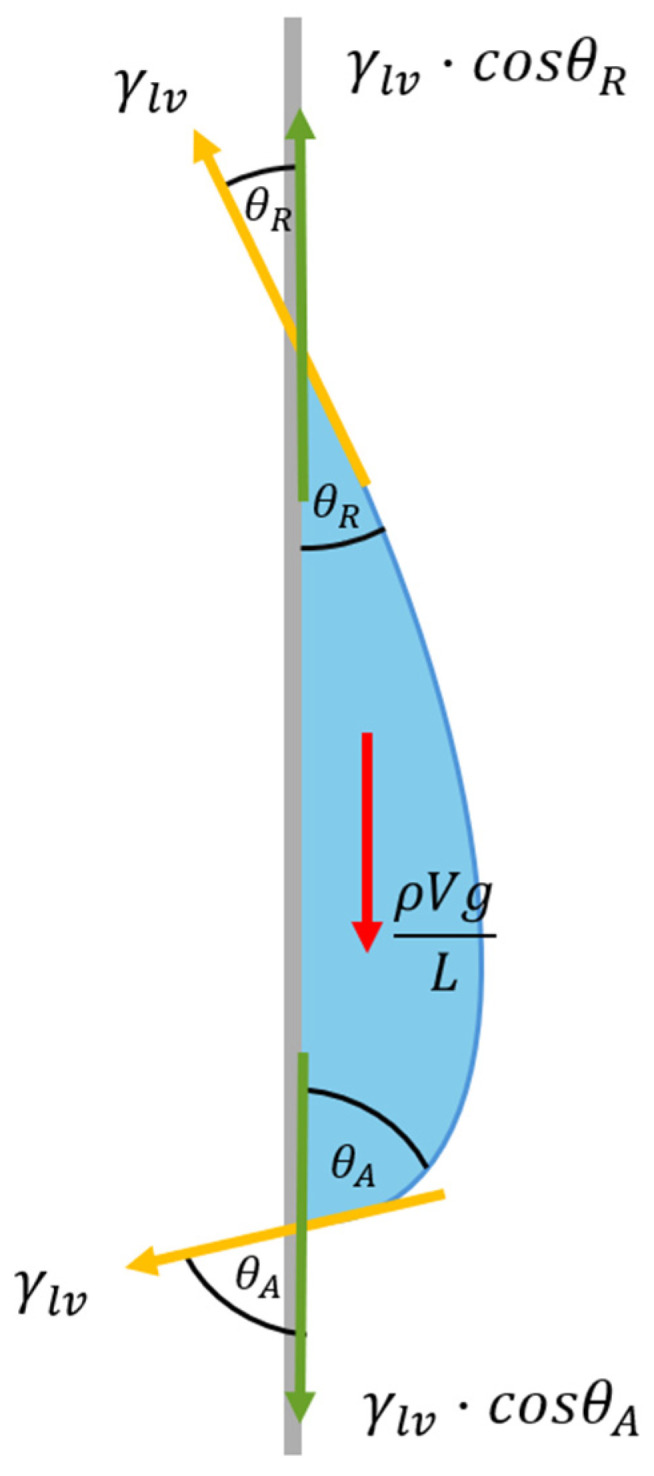
The diagram showing forces acting on a droplet resting on a substrate.

The movement of droplets during condensation on a surface is governed by fundamental physical principles, primarily involving the interplay among contact line, contact angle hysteresis, and droplet volume. As illustrated in Figure 7, two primary forces act on a droplet resting on a substrate: the gravitational force, which drives the droplet downward, and the resistance force, which pins the droplet to the surface. According to Equation (1), the gravitational force promoting droplet movement and detachment is directly influenced by the droplet volume (*V*). In contrast, the resistance force depends on the contact line length (*L*), the surface tension ($\gamma_{lv}$), the advancing contact angle ($\theta_{A}$), and the receding contact angle ($\theta_{R}$) [45]. It is assumed that gravitational acceleration (g) and the droplet density (ρ) remain constant.

(1) $$\frac{\rho Vg}{L}\;=\;\gamma_{lv}\;\cdot \left(cos\theta_{R}-cos\theta_{A}\right)$$

Contact angle hysteresis refers to the difference between the advancing and receding contact angles. In Equation (1), the term $cos\theta_{R}-cos\theta_{A}$ reflects this behavior: when the difference between the advancing and receding angles is small, the difference in their cosine values is also small. Conversely, a larger angular difference results in a greater cosine difference. Surface tension, contact angle hysteresis, and gravitational force collectively determine whether a droplet stays on or detaches from a surface. Both surface tension and contact angle, which influence droplet adhesion, are highly dependent on the surface morphology and the type of liquid. Additionally, the length of the contact line is affected by the contact angle of the droplet. As water contact angle decreases, the droplet spreads and flattens, increasing the contact line. A droplet will begin to move when the gravitational force—proportional to its volume—exceeds the opposing force arising from surface tension and contact angle hysteresis.

Since each surface in this research exhibited different contact angles, contact line length, and contact angle hysteresis values, the droplet volume required for detachment also varied. The unpolished surface demonstrated the hydrophobic characteristics, where condensation primarily occurred in the form of dropwise condensation. The adhesion between the droplet and the surface was substantial, as indicated by the value of $cos\theta_{R}-cos\theta_{A}$, which reflects a strong resistance to the downward movement of the droplet. This high resistance prevented the droplet from detaching easily. As the droplets grew and coalesced with nearby droplets, the downward force—influenced by the increasing volume of the droplets—gradually increased. Once this force exceeded the resistance force due to surface tension, contact line length, and the contact angle hysteresis, the droplets began to slide down the surface, sweeping up droplets in their path. However, many small droplets remained pinned to the surface for an extended period without detaching, which limited the availability of free surface area for further condensation. As a result, the unpolished surface collected a smaller amount of water compared to the polished, lasered and coated, and lasered-patterned surfaces. In addition to the unpolished surface, the polished surface exhibited moderate hydrophilic characteristics, with a water contact angle of 77.7 ± 1.2°. Due to a slight increase in the contact angle hysteresis and a longer contact line, the polished surface exhibited a higher resistance force when compared to the unpolished surface. However, the extended contact line also caused the droplets to spread more, promoting coalescence. Consequently, a greater volume of water accumulated on the surface, allowing more water to be harvested over at the same period. This phenomenon contributed to the enhanced water harvesting performance of the polished surface compared to the unpolished surface. Apart from the unpolished and polished surfaces, the coated surface exhibiting superhydrophobic characteristics, with a high contact angle of 157.0 ± 1.5° and a very small contact angle hysteresis of less than 10°. As a result, the coated surface demonstrated high water-repellent capability. Condensation on this surface occurred via dropwise condensation. The extremely high contact angle caused droplets to form nearly spherical shapes and jump from the surface almost immediately. However, this rapid and random detachment hindered the accumulation of larger droplets, leading to reduced water harvesting performance. As shown in the results, the amount of condensed water collected from the coated surface was the lowest at 101.3 mL/m^2^. These findings indicate that while superhydrophobic surfaces are beneficial for self-cleaning and heat transfer enhancement applications, they are less suitable for water harvesting, where droplet retention and accumulation are essential for optimal performance. In contrast to the dropwise condensation observed on the coated surface, the lasered surface with the low contact angle of 35.9 ± 1.4° exhibited strong hydrophilic property. With the small contact angle hysteresis, the resistance force exerting droplets on the lasered surface declined. In addition, its extended contact line facilitated rapid droplet coalescence and growth. As the volume increased, the gravitational force more readily overcame the weak resistance force, enabling timely detachment. A maximum water harvesting rate of the lasered surface of 386.7 mL/m^2^ was recorded. Both polished and lasered surfaces exhibited hydrophilic characteristics; however, the lower contact angle of the lasered surface promoted greater droplet spreading and more efficient coalescence, leading to enhanced water harvesting performance compared to the polished surface [46]. The coated and lasered patterned surfaces, combining superhydrophobic and hydrophilic regions, exhibited a complex condensation process. The hydrophilic areas promoted droplet coalescence and growth, while the superhydrophobic regions facilitated droplet detachment and movement. As droplets from adjacent hydrophilic regions grew large enough, they coalesced and spread over the superhydrophobic regions due to their water-repellent properties. The superhydrophobic surface reduced the adhesion between the droplet and the surface, facilitating faster droplet merging from nearby hydrophilic regions. As the volume of the merging droplet increased, the gravitational force also increased. This interaction between hydrophilic and superhydrophobic areas enhanced water harvesting by promoting both droplet growth and quick detachment. Among all biphilic surfaces in this study, it was found that the 1:4 coated and lasered patterned surface demonstrated the best water harvesting performance with the total water harvesting amount of 340 mL/m^2^. Further investigation revealed that the optimal water harvesting occurred at the widths of 0.5 mm for the superhydrophobic area and 2 mm for the hydrophilic area. When the width of the coated region increased, the water harvesting performance declined because the superhydrophobic strip was too large. This reduced the effectiveness of the merging process, as droplets forming near the hydrophilic regions were less able to coalesce efficiently. On the other hand, when the width of the coated region was reduced, the width of the hydrophilic region also decreased. Since the hydrophilic area played a crucial role in promoting droplet growth, reducing its size delayed droplet formation and growth, resulting in lower water harvesting performance.

## 5. Conclusions

In this study, we explored the water harvesting performance of the modified nanostructured aluminum surfaces. Five types of surfaces were fabricated: unpolished, polished, coated, lasered, and coated and lasered patterned. The polished sample was prepared using the sandpaper polishing method. Silica nanoparticles coating was used to fabricate the coated sample. The nanolaser ablation was performed to fabricate the lasered sample. The coated and lasered patterned samples with various coated-to-lasered area ratios including 1:1, 1:2, 1:3, 1:4, 1:5, and 1:6 were produced using both coating and nanolaser fabrication methods. The coated region was fixed at 0.5 mm, while the lasered region varied from 0.5 to 3 mm accordingly. The surface wettability tests showed that the unpolished surface was hydrophobic, the polished surface was hydrophilic, the coated surface was superhydrophobic, the lasered surface was strong hydrophilic, and the coated and lasered patterned surfaces exhibited both superhydrophobic and hydrophilic characteristics. The surface wettability, morphology, and EDS analysis were also conducted. Key findings showed that the lasered sample achieved the best water harvesting performance at 386.7 mL/m^2^, representing improvements of 42% and 282% compared to the unpolished and coated samples, respectively. The polished and the 1:4 coated and lasered patterned samples demonstrated strong performance, with water harvesting values of 342.7 mL/m^2^ and 340 mL/m^2^, respectively, marking increases of over 20% and 230% when compared with the unpolished and coated samples. Among all coated and lasered patterned configurations, the 1:4 coated and lasered patterned ratio with the superhydrophobic width of 0.5 mm and the hydrophilic width of 2 mm yielded the highest water harvesting performance. In addition to experimental results, the study analyzed the roles of surface tension, contact angle hysteresis, and contact line in impacting droplet behavior on the surface. These findings provide foundational insights into how surface structure influences condensation-based water harvesting. This study can potentially open new avenues for future research, particularly in optimizing surface patterns and configurations to maximize water harvesting performance under diverse environmental conditions. In future studies, a broader range of surface pattern configurations to further improve water harvesting performance will be explored. Ultimately, these surface design configurations can be scaled up for practical implementation under real-world climatic conditions.

## Figures and Tables

**Table 1:** Measured surface parameters of the prepared surfaces.

Surface Parameters	Unpolished	Polished	Coated	Lasered
Contact Angle (°)	94.1 ± 1.0	77.7 ± 1.2	157.0 ± 1.5	35.9 ± 1.4
Surface Energy (mN/m)	21.1 ± 1.9	33.5 ± 0.8	0.4 ± 0.1	78.9 ± 1.3
Surface Roughness (μm)	6.3 ± 0.3	5.1 ± 0.1	5.5 ± 0.1	4.8 ± 0.1
Advancing Contact Angle (°)	95.3 ± 0.2	89.0 ± 0.4	152.9 ± 1.4	39.0 ± 0.4
Receding Contact Angle (°)	77.7 ± 0.4	68.5 ± 0.4	145.9 ± 1.1	34.4 ± 0.1
Contact Angle Hysteresis (°)	17.7 ± 0.2	20.5 ± 0.2	6.9 ± 0.3	4.5 ± 0.3

## Data Availability

The data presented in this study are available upon request.
